# Involvement of jasmonic acid, ethylene and salicylic acid signaling pathways behind the systemic resistance induced by *Trichoderma longibrachiatum* H9 in cucumber

**DOI:** 10.1186/s12864-019-5513-8

**Published:** 2019-02-18

**Authors:** Min Yuan, Yuanyuan Huang, Weina Ge, Zhenhua Jia, Shuishan Song, Lan Zhang, Yali Huang

**Affiliations:** 1 College of Life Sciences, North China University of Science and Technology, Tangshan, 063210 People’s Republic of China; 2 Biology Institute, Hebei Academy of Sciences, Shijiazhuang, 050081 People’s Republic of China

**Keywords:** Cucumber, *Trichoderma longibrachiatum*, *Botrytis cinerea*, Transcriptome, Proteome, Defense response, Phytohormones, Secondary metabolites

## Abstract

**Background:**

*Trichoderma* spp. are effective biocontrol agents for many plant pathogens, thus the mechanism of *Trichoderma*-induced plant resistance is not fully understood. In this study, a novel *Trichoderma* strain was identified, which could promote plant growth and reduce the disease index of gray mold caused by *Botrytis cinerea* in cucumber. To assess the impact of *Trichoderma* inoculation on the plant response, a multi-omics approach was performed in the *Trichoderma*-inoculated cucumber plants through the analyses of the plant transcriptome, proteome, and phytohormone content.

**Results:**

A novel *Trichoderma* strain was identified by morphological and molecular analysis, here named *T. longibrachiatum* H9. Inoculation of *T. longibrachiatum* H9 to cucumber roots promoted plant growth in terms of root length, plant height, and fresh weight. Root colonization of *T. longibrachiatum* H9 in the outer layer of epidermis significantly inhibited the foliar pathogen *B. cinerea* infection in cucumber. The plant transcriptome and proteome analyses indicated that a large number of differentially expressed genes (DEGs) and differentially expressed proteins (DEPs) were identified in cucumber plants 96 h post *T. longibrachiatum* H9 inoculation. Up-regulated DEGs and DEPs were mainly associated with defense/stress processes, secondary metabolism, and phytohormone synthesis and signaling, including jasmonic acid (JA), ethylene (ET) and salicylic acid (SA), in the *T. longibrachiatum* H9-inoculated cucumber plants in comparison to untreated plants. Moreover, the JA and SA contents significantly increased in cucumber plants with *T. longibrachiatum* H9 inoculation.

**Conclusions:**

Application of *T. longibrachiatum* H9 to the roots of cucumber plants effectively promoted plant growth and significantly reduced the disease index of gray mold caused by *B. cinerea*. The analyses of the plant transcriptome, proteome and phytohormone content demonstrated that *T. longibrachiatum* H9 mediated plant systemic resistance to *B. cinerea* challenge through the activation of signaling pathways associated with the phytohormones JA/ET and SA in cucumber.

**Electronic supplementary material:**

The online version of this article (10.1186/s12864-019-5513-8) contains supplementary material, which is available to authorized users.

## Background

*Botrytis cinerea* is a necrotrophic fungal pathogen that infects more than 200 commercial crops, leading to significant agricultural losses worldwide [1]. To date, chemical fungicides remain the primary method of controlling this pathogen. Due to the detrimental effects on the environment and potential risks for human health of chemical fungicides, a biocontrol method would constitute a preferable alternative approach [2]. *Trichoderma* spp. are plant growth-promoting fungi that have been found to have great potential for the control of gray mold caused by *B. cinerea* [3, 4]. The biocontrol can be achieved by a direct interaction between *Trichoderma* and pathogens, or indirectly by inducing plant resistance to pathogens in the same or different parts from the pathogen infection sites. Traditionally, *Trichoderma* spp. directly antagonize pathogens by mycoparasitism, secretion of antibiotics, and competition for space and nutrients [5–7]. However, subsequent discoveries have demonstrated that these biocontrol agents can also interact intimately with plant roots, even colonizing the outer epidermal layers, and act as avirulent, opportunistic plant symbionts [3, 7]. Root colonization by selected *Trichoderma* isolates has been reported to promote plant growth and increase resistance to a variety of pathogens, both soil-borne and foliar, in various plant species [8–12]. Although the mechanism of induced plant resistance is not fully understood, the plant response to *Trichoderma* spp. has been studied at the physiological and biochemical levels. These studies have shown that colonization by *Trichoderma* spp. triggers a series of plant responses that may enhance the defensive capacity of the plant. Mitogen-activated protein kinase (MAPK) cascades are activated in *Arabidopsis thaliana* leaves by inoculation of the roots with *T. hamatum* T382 [8]. The cucumber MAPK homologous to *A. thaliana* MPK3 is induced by incubation of the roots with *T. asperellum* T203 [13]. Activation of MAPK cascades will alter the phosphorylation status of many substrate proteins to regulate diverse downstream signaling pathways, such as phytohormone signaling [14]. Plant hormone signaling may modulate the defense network by translating *Trichoderma*-induced upstream signaling events into the activation of defense responses.

In the past, researchers found that contacting with pathogenic and non-pathogenic organisms would trigger two main defense mechanisms in plants: systemic acquired resistance (SAR) and induced systemic resistance (ISR). SAR is commonly triggered by local pathogen attack, correlating with the involvement of salicylic acid (SA) and the activation of PR genes. ISR is activated when the plant interacts with non-pathogenic rhizosphere fungi or bacteria, and requires jasmonic acid (JA)/ethylene (ET) [10, 15]. However, the traditional knowledge of ISR became controversial in recent years. Increasing research has demonstrated that *Trichoderma* may induce systemic resistance through multiple hormonal signaling pathways, and the specific hormones involved are dependent on both the plant species and *Trichoderma* strains involved. The cross-talk among different hormone signaling pathways may render plants capable of finely regulating immune responses [8, 10, 15–23]. It has also been demonstrated that *Trichoderma* spp. can positively interfere with reactive oxygen species (ROS) production, e.g., through increasing the ROS scavenging ability of the plant at future infection sites, thereby potentially reducing the damaging levels of ROS in the leaves, thus limiting *B. cinerea* infection [24–27]. The expression of defense-related proteins can be strongly potentiated when plants are challenged by additional pathogens, even at sites distant from the location of *Trichoderma* colonization [3]. Along with other defense strategies, the production of secondary metabolites, such as phytoalexins, plays important roles in the plant defense against *B. cinerea* [26, 28–32]. Collectively, it is likely that *Trichoderma* spp. induce multiple levels of changes in plant metabolism that can lead to increased resistance to abiotic and biotic stresses, and thus the mechanism of *Trichoderma*-induced systemic resistance requires further investigation [33].

Cucumber is one of the most economically important vegetables worldwide. It is susceptible to infection by the plant-pathogenic fungus *B. cinerea*. Finding potential biocontrol *Trichoderma* spp. against cucumber diseases is thus warranted [34, 35]. In this study, a novel *Trichoderma* strain was identified, namely *T. longibrachiatum* H9, which could promote plant growth and reduce the disease index of gray mold caused by *B. cinerea* in cucumber. Furthermore, the effect of *T. longibrachiatum* H9 on induction of defense response in cucumber plants was studied through the analyses of the plant transcriptome, proteome and phytohormone content. The large-scale, multi-omics datasets allowed us to monitor changes in cucumber gene and protein expressions and also provided a basis from which to illustrate the biocontrol mechanism of *T. longibrachiatum* H9 induced systemic resistance against *B. cinerea*.

## Methods

### Morphological and molecular identification of the novel strain

The *Trichoderma* H9 strain was grown on potato dextrose agar (PDA) medium at 30 °C and identified by morphological observation based on the monograph of Gams and Bisset [36]. The spores were harvested by flooding the surface of the cultures with distilled water. One milliliter of spore suspension was aliquoted and inoculated into potato-dextrose (PD) broth in a rotary shaker at 30 °C and 200 rpm for 48 h. The mycelium was collected by filtration and washed three times with distilled water. Genomic DNA was extracted using a commercial DNA extraction kit. Using the *Trichoderma* strain H9 genome as the template, the ITS and TEF-1α genes were amplified using specific primers, which were described in Additional file 1: Table S1 (ITS4 and ITS6 for ITS, as well as EF1-728F and TEF1-LLErev for TEF-1α). The PCR products were then sequenced by the Sangon Biotechnology Co., Ltd. Shanghai, China. The ITS and TEF-1α sequences of this strain were deposited in GenBank with the accession numbers MF111118 and MK179408 respectively, and were then aligned with the corresponding sequences of known *Trichoderma* strains using NCBI BLAST. The phylogenetic trees were constructed under Maximum Likelihood method using MEGA 7 software [37] based on the ITS or TEF-1α sequences. The phylogeny tests were evaluated through performing 1000 bootstrap replicates.

### Plant growth, *Trichoderma* inoculation and pathogen challenge

Seeds of cucumber (*Cucumis sativus* L.) were purchased from Tianjin Kernel Cucumber Research Institute (Tianjin, China). The seeds were sterilized in 70% ethanol for 2 min, 2.0% NaOCl for 2 min, washed with sterile water three times, and then placed on a sterile gauze sheet with liquid ^1^/_2_ Murashige and Skoog (MS) medium in a Petri dish at 37 °C for 24 h. Germinated seeds (30 per box) were placed on a sterile gauze sheet on top of a sterile steel screen in a 50 × 25 × 8 (depth) cm pot system under a 12 h light/12 h dark cycle at 25 ± 2 °C with a light intensity of 120 μmol/m^2^s. The steel screen holds the seeds 1 cm above the liquid medium [38].

*Trichoderma* inoculation was performed when the plants had grown to the 3-leaf stage, at which point the spore suspension of *Trichoderma* H9 at a final concentration 1 × 10^6^ cfu/mL was added to the pot. ^1^/_2_ MS medium was used instead of spore suspension in the control group.

The *B. cinerea* challenge was performed 24 h post H9 inoculation (24 hpTi). Ten microliters of *B. cinerea* spore suspension at a concentration of 1 × 10^8^ cfu/mL was injected into the abaxial surface of the cucumber leaves. Water was injected as the control for the *B. cinerea* challenge. In total, four different treatment groups were established, namely non-inoculated, non-challenged plants (−T-B), non-inoculated, challenged plants (−T + B), inoculated, non-challenged plants (+T-B), and inoculated, challenged plants (+T + B) [10].

### Detection of promoting effect on plant growth

The method for *Trichoderma* plant inoculation was described above. Thirty plants were used for each treatment. Treated plants were placed under a 12 h light/12 h dark cycle at 25 ± 2 °C. After two weeks, the plants were harvested and the root length, plant height and fresh weight were measured. Three replicates were performed.

### Detection of biocontrol efficacy

The method for *Trichoderma* plant inoculation and *B. cinerea* challenge was described above in the -T + B and + T + B groups. Three different sites per leaf were selected on the second and third leaves of each plant. Twelve plants for each group were treated. Three replicates were performed. Following treatment, the cucumber plants were grown under a 12 h light/12 h dark cycle at 25 ± 2 °C. The disease severity of the gray mold was recorded daily. When *B. cinerea* broadly infected the cucumber leaves in the -T + B group, the disease index and inhibition rate were calculated according to the standard for gray mold disease progression. The disease progression of the infected plants was divided into five grades, where grade 0 = no visible gray mold spot, grade 1 = 1–5%, grade 3 = 6–15%, grade 5 = 16–25%, grade 7 = 26–50%, and grade 9 = ≥51% of the leaf surface covered with gray mold. The gray mold development on each plant was recorded using the above 1–5 scale, and the disease index was calculated using the following formula [39]:

$$ \mathrm{Disease}\ \mathrm{in}\mathrm{dex}=\Sigma\ \left(\mathrm{number}\ \mathrm{of}\ \mathrm{leaves}\ \mathrm{in}\ \mathrm{each}\ \mathrm{disease}\ \mathrm{grade}\times \mathrm{grade}\mathrm{s}\ \mathrm{value}\right)/\left(\mathrm{total}\ \mathrm{number}\ \mathrm{of}\ \mathrm{assessed}\ \mathrm{leaves}\times \mathrm{the}\ \mathrm{highest}\ \mathrm{grade}\ \mathrm{value}\right) $$

$$ \mathrm{Disease}\ \mathrm{inhibition}\ \mathrm{rate}\ \left(\%\right)=\left(\mathrm{disease}\ \mathrm{index}\ \mathrm{of}\ \mathrm{control}-\mathrm{disease}\ \mathrm{index}\ \mathrm{after}\ \mathrm{treatment}\right)/\mathrm{disease}\ \mathrm{index}\ \mathrm{of}\ \mathrm{control}\times 100 $$

### Sample processing for transmission electron microscopic study

Cucumber root samples (4 to 10 mm) were collected from the main root at the *Trichoderma* penetration sites, 96 hpTi. Samples were treated and then examined in a Hitachi H-7650 transmission electron microscope as described [31, 38]. The sites of root colonization by *Trichoderma* were characterized by comparing the samples from *Trichoderma*-inoculated and non-inoculated groups. Five samples from five different roots for each group, and 10 ultrathin sections for each sample were observed.

### Statistical analysis

Statistical analysis was performed using SPSS software, version 20.0 (SPSS, Inc., Chicago, IL, US). The data from plant growth promoting assays, biocontrol efficacy and plant hormones measurement assays were analyzed by one-way analysis of variance (ANOVA), and the statistical significance was determined using pair-wise comparisons (*P* < 0.05).

### Transcriptome analysis by RNA-Seq

The second and third cucumber leaves were collected from the -T-B and + T-B groups at 96 hpTi respectively when the plants in the +T + B group showed a clear inhibition of *B. cinerea* infection. The samples were immediately frozen in liquid nitrogen, and then sent to Shanghai Biotechnology Co., Ltd. Shanghai, China for RNA sequencing. The reference genome for transcriptome analysis was acquired from ftp://cucurbitgenomics.org/pub/cucurbit/genome/cucumber/Chinese_long/v2/. Three biological replicates in each group were performed and raw data were deposited into the Sequence Read Archive database with the accession number SRP136435. A basic analysis was performed on the transcriptome data, including data pre-processing, genomic mapping, gene expression analysis, analysis of differentially expressed genes (DEGs), and Gene Ontology (GO)/Kyoto Encyclopedia of Genes and Genomes (KEGG) enrichment analysis of DEGs. Gene expression levels were determined by FPKM (Fragments Per Kilobase of exon model per Million mapped reads) as described [40]. In order to screen DEGs, up- and down-regulation of genes was defined when the adjusted *P*-value was < 0.05 and the absolute fold change was ≥2.0 (∣log_2_^(fold change)^∣ ≥ 1.0). Regarding the GO/KEGG enrichment analysis, *P*-value < 0.05 was chosen as the standard to determine enrichment.

### Translatome analysis by iTRAQ mass spectrometry

The second and third cucumber leaves were collected from the -T-B and + T-B groups at 96 hpTi, and immediately frozen in liquid nitrogen, respectively, and then sent to Allwegene Technology Inc. Beijing, China for iTRAQ sequencing. Raw data related to this study have been deposited into iProX (ww.iprox.org) with ID IPX0001185000/PXD009314. Protein identification was performed using the ProteinPilot™ v4.5 search engine against a database (Uniprot *Cucumis sativus* L). Only peptides with an unused score ≥ 1.3 and confidence interval > 95% were counted to reduce the probability of false peptide identification. Each confident protein was identified with at least one unique peptide. Furthermore, differentially expressed proteins (DEPs) were screened by iTRAQ quantification and up- and down-regulation of proteins was defined when the *P*-value was < 0.05 and the absolute fold change was ≥2.0. GO/KEGG enrichment analyses of differentially expressed proteins (DEPs) for functional annotations were further performed, and *P*-value < 0.05 was selected as the standard to determine the enrichment.

### Correlation analysis between transcriptome and proteome data

The expression trends of the genes from the transcriptome analysis and their corresponding proteins from the proteome analysis were compared as described [41]. The results were divided into three categories: correlated genes/Proteins, DEGs/DEPs with corresponding proteins/genes, and DEGs/DEPs with corresponding DEPs/DEGs. Correlated DEGs/DEPs were further divided into two categories: with the same expression trend and the opposite expression trend.

### Plant hormones measurement

The same treatment groups were established (−T-B and + T-B) as in the previous experiments. The cucumber leaves were harvested from each group at 96 hpTi, and the SA and JA contents were measured using HPLC/MS/MS (high performance liquid chromatography tandem mass spectrometry), which was performed by Zoonbio Biotechnology Co., Ltd. Nanjing, China. Samples were separated by Agilent1290 Infinity liquid chromatography from Agilent (Santa Clara, CA, US) and analyzed by QTrap 6500 mass spectrometer from AB Sciex (Framingham, MA, US) as described [42]. Three replicates were performed.

### Quantitative real-time (qRT-PCR) analysis marker gene expression

Leaf tissue was collected from the second and third cucumber leaf in the -T-B and + T-B groups at 96 hpTi, respectively. Total RNA was extracted from each sample using RNAiso™ Plus reagents according to the manufacturer’s instructions. The cDNA synthesis and qRT-PCR were performed according to the PrimeScript™ RT reagent kit. Specific primers used in the qRT-PCR were described in Additional file 1: Table S1. Expression values were normalized to the *ACT2* gene. The experiments were repeated three times.

## Results

### Morphological and molecular identification of the *Trichoderma* H9 strain

The *Trichoderma* H9 strain was obtained from the soil near a cucumber plant and exhibited the typical morphological features of *T. longibrachiatum* based on the monograph of Gams and Bisset [36]. Molecular identification based on ITS and TEF-1α genes revealed that this H9 strain shared the highest homology in the phylogeny and clustered together with *T. longibrachiatum* strains, whose species identity has been confirmed by phylogenetic analysis [43–47]. The H9 strain was thus identified to be *T. longibrachiatum*, and was accordingly named *T. longibrachiatum* H9.

### Plant growth enhancement by *T. longibrachiatum* H9 inoculation in cucumber plants

Furthermore, growth-promoting effects by *T. longibrachiatum* H9 inoculation were observed based on root length, plant height, and fresh weight measurements of the cucumber plants. The average root length, plant height, and fresh weight of the *T. longibrachiatum* H9-inoculated plants were 7.46 ± 0.35 cm, 3.79 ± 0.07 cm, and 48.63 ± 1.88 g, which were significantly higher than those of untreated plants with 5.02 ± 0.16 cm, 3.42 ± 0.11 cm, and 38.16 ± 1.53 g, respectively (*P* < 0.05). The *T. longibrachiatum* H9 strain significantly impacted root length (48.68%), plant height (10.82%), and fresh weight (27.44%) in cucumber (Table 1).

**Table 1:** Inoculation of *T. longibrachiatum* H9 to cucumber roots effectively promoted cucumber plant growth

Strains	Root length (cm)	Increase rate (%)	Plant height (cm)	Increase rate (%)	Fresh weight (g)	Increase rate (%)
H9	7.46 ± 0.35 *	48.68	3.79 ± 0.07 *	10.82	48.63 ± 1.88 *	27.44
Control	5.02 ± 0.16	–	3.42 ± 0.11	–	38.16 ± 1.53	–

Note: * and ** indicate significance at *P* < 0.05 and 0.01

### Biocontrol effects by *T. longibrachiatum* H9 against *B. cinerea*

In order to determine if *T. longibrachiatum* H9 could reduce the symptoms of *B. cinerea* infection in cucumber, 3-leaf stage cucumber plant roots were treated with *T. longibrachiatum* H9 and their susceptibility to *B. cinerea* (+T + B) leaf infection was subsequently assessed by comparison to mock-treated plants (−T + B). Treatment of cucumber with *T. longibrachiatum* H9 significantly reduced the disease index after 4, 8, and 12 d, and the inhibition rates increased to 56.7% on the 12th day from 49.62% on the 4th day (*P* < 0.05; Table 2).

**Table 2:** Treatment of cucumber with *T. longibrachiatum* H9 significantly reduced the disease index of gray mold

Groups	Days after H9 inoculation					
	4 d		8 d		12 d	
	Disease index	Inhibition rate%	Disease index	Inhibition rate%	Disease index	Inhibition rate%
-T + B	0.339 ± 0.028	–	0.465 ± 0.042	–	0.530 ± 0.011	–
+T + B	0.267 ± 0.036*	49.62	0.234 ± 0.016*	55.85	0.213 ± 0.017*	56.7

Note: * and ** indicate significance at *P* < 0.05 and 0.01

**Fig. 1:**
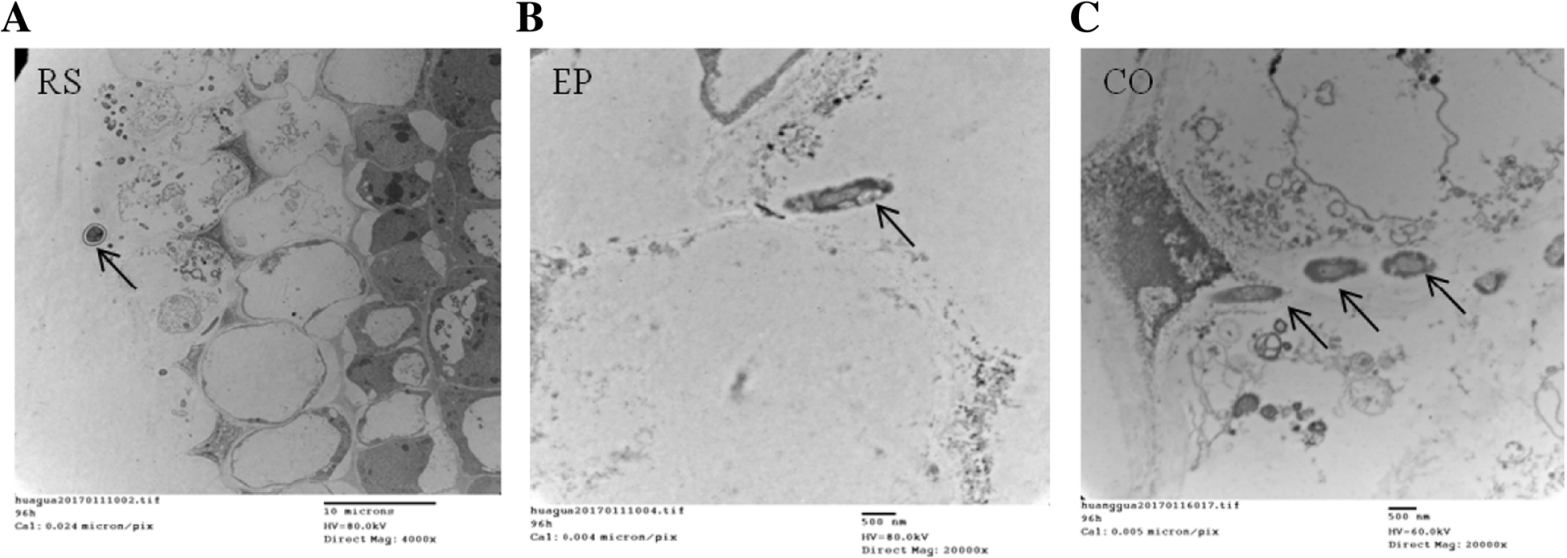
Transmission electron micrographs indicated that *T. longibrachiatum* H9 had colonized the cucumber roots. *Trichoderma* hyphae developed at the root surface **(a)**, penetrated into the root epidermis **(b)**, and progressed towards the cortex **(c)**. Arrows indicate *Trichoderma* hyphae. Bars: A, 10 μm; B, 500 μm; C, 500 μm

Consistently, *T. longibrachiatum* H9 penetrated cucumber root cortical cells and was restricted in the out layer of epidermis after 96 h (96 hpTi), which allowed sufficient time for spore germination, colonization of the roots and induction of a plant response. A large number of *Trichoderma* hyphae developed at the root surface (RS) (Fig. 1a), penetrated into the root epidermis (EP) (Fig. 1b) and progressed towards the cortex (CO) (Fig. 1c), mainly by intercellular growth. The mycelia of *T. longibrachiatum* H9 grew mainly between the cells and thickened the cell wall without entering the cell, as observed by transmission electron microscopy. Taken together, the biocontrol assays indicated that root colonization by *T. longibrachiatum* H9 may induce systemic disease resistance against the foliar pathogen *B. cinerea* in cucumber.

### Deciphering the plant responses associated with *T. longibrachiatum* H9 inoculation using transcriptome analysis and iTRAQ proteomics

To examine the underlying mechanisms of the plant response associated with *T. longibrachiatum* H9-induced plant resistance, both RNA-Seq and proteome analysis were performed on cucumber plant samples harvested at 96 hpTi from the -T-B and + T-B groups, respectively. Compared to untreated plants (+T-B vs. -T-B), 3765 DEGs were identified from the RNA-Seq analysis, with 2304 up-regulated genes and 1461 down-regulated genes under *T. longibrachiatum* H9 inoculation. A total of 922 DEPs were identified from the proteome analysis with 428 up-regulated and 494 down-regulated proteins (*P* < 0.05 and fold change > 2.0; Table 3). GO enrichment and KEGG enrichment analyses were further performed to classify the up-regulated and down-regulated DEGs and DEPs.

**Table 3:** Summary of the number of DEGs and DEPs detected in the *T. longibrachiatum* H9-inoculated cucumber plants, compared to untreated plants (+T-B vs. -T-B)

	Genes	Proteins
Total DEGs/DEPs	3765	922
Up-regulated	2304	428
Down-regulated	1461	494

Based on up-regulated DEGs or DEPs, biological processes was the most enriched category and the GO terms related to phytohormones, secondary metabolism and defense/stress processes were significantly enriched (*P* < 0.05). The biosynthesis and signaling of hormones, including JA/ET and SA, were observed as plant responses in the plant hormones category. “Phenylpropanoid biosynthetic process”, “flavonoid biosynthetic process”, “lignin biosynthetic process” and other secondary metabolic processes were identified in the secondary metabolites category. Many GO terms related to defense/stress processes were observed including “defense response”, “response to oxidative stress”, “response to reactive oxygen species”, and so on. Meanwhile, several signal transduction GO terms were observed, such as “MAPK cascade” and “signal transduction by protein phosphorylation” (*P* < 0.05; Additional file 2: Table S2, Additional file 3: Table S3). Based on down-regulated DEGs or DEPs, “photosynthesis” and “photosynthesis, light reaction” were significantly enriched terms (*P* < 0.05; Additional file 4: Table S4, Additional file 5: Table S5).

**Fig. 2:**
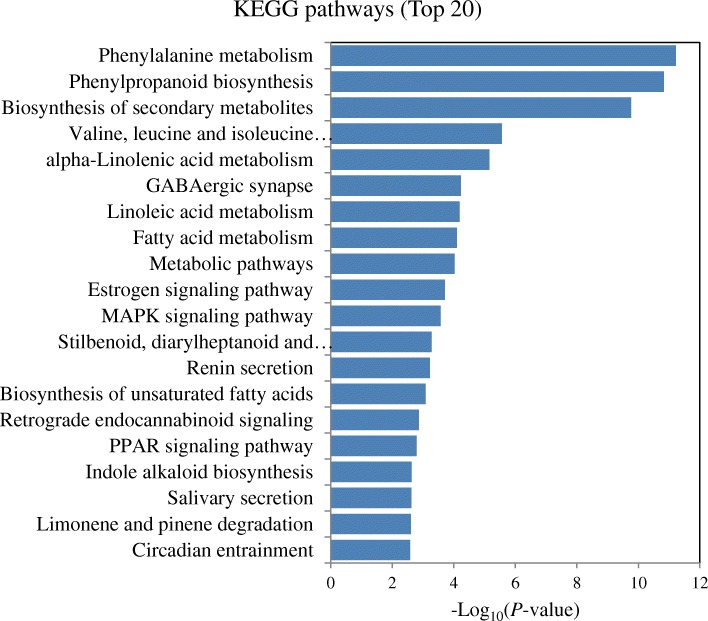
The top 20 enriched KEGG pathways based on up-regulated DEGs in the *T. longibrachiatum* H9-inoculated plants in comparison to untreated plants (+T-B vs.-T-B)

**Fig. 3:**
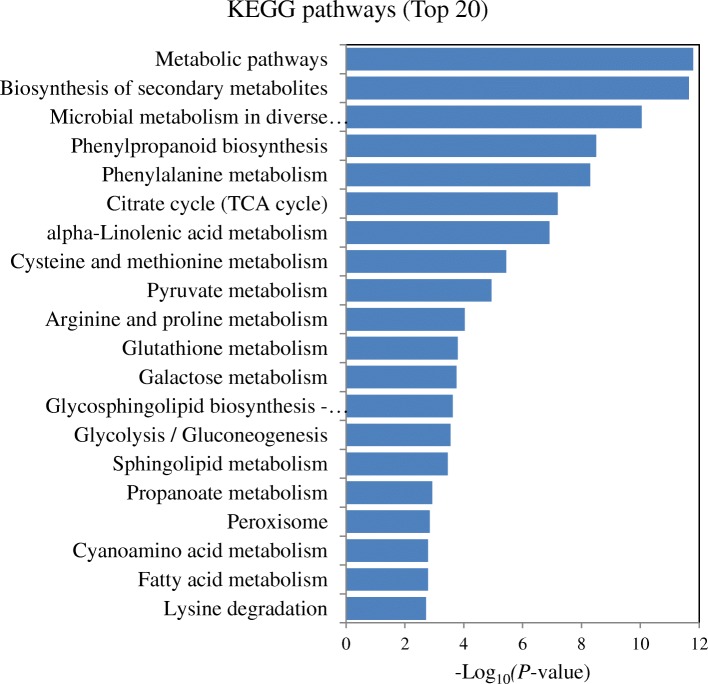
The top 20 enriched KEGG pathways based on up-regulated DEPs in the *T. longibrachiatum* H9-inoculated plants in comparison to untreated plants (+T-B vs.-T-B)

The KEGG enrichment analysis allowed us to identify specific pathways according to DEGs or DEPs, respectively. The pathways related to the biosynthesis of phytohormones, including “phenylalanine metabolism (SA biosynthesis)”, “alpha-linolenic acid metabolism (JA biosynthesis)”, and “cysteine and methionine metabolism (ET biosynthesis)”, were significantly enriched based on up-regulated DEGs or DEPs, respectively. Moreover, the pathways “phenylpropanoid biosynthesis”, “flavonoid biosynthesis”, “MAPK signaling pathway” and “peroxisome pathway”, which are associated with secondary metabolite biosynthesis, signal transduction, or defense/stress processes, were also activated in the *T. longibrachiatum* H9-inoculated cucumber plants (*P* < 0.05; Additional file 6: Table S6, Additional file 7: Table S7). The top 20 enriched KEGG pathways based on up-regulated DEGs or DEPs were shown, respectively (Figs. 2 and 3). The presence of *T. longibrachiatum* H9 reprogrammed the gene and protein expression related to phytohormones, secondary metabolism, and defense/stress processes in cucumber plants. This suggested that *T. longibrachiatum* H9 inoculation might enhance plant defense capacity through activating the signaling pathways associated with the phytohormones JA/ET and SA.

### Correlation analysis of transcriptome and proteome data

**Fig. 4:**
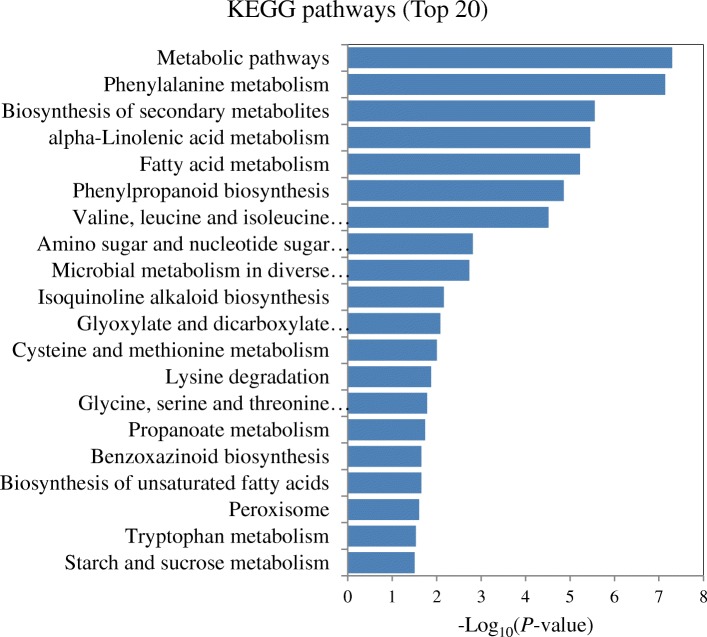
The top 20 enriched KEGG pathways based on up-regulated corresponding DEGs/DEPs in the *T. longibrachiatum* H9-inoculated plants in comparison to untreated plants (+T-B vs.-T-B)

To further explore the findings from both transcriptome and proteome data, the correlation analysis was performed by comparing the expression trends of the genes from the transcriptome analysis and their corresponding proteins from the proteome analysis. 3938 genes/proteins were correlated in the totally detected 4171 proteins and 20,189 genes. Among the 3765 DEGs/922 DEPs, 798 DEGs/835 DEPs showed corresponding proteins/genes, and 277 DEGs/DEPs showed corresponding DEPs/DEGs. Furthermore, 241 DEGs/DEPs showed the same expression trends with 132 DEGs/DEPs up-regulated and 109 DEGs/DEPs down-regulated (Table 4, Additional file 8: Table S8). The enriched KEGG pathways based on the 132 up-regulated DEGs/DEPs were analyzed and the top 20 pathways were shown (Fig. 4). “Phenylalanine metabolism (SA biosynthesis)”, “alpha-linolenic acid metabolism (JA biosynthesis)”, “cysteine and methionine metabolism (ET biosynthesis)”, “biosynthesis of secondary metabolites”, “phenylpropanoid biosynthesis”, and “peroxisome” pathways were remain significantly enriched. In contrast, “ribosome”, “porphyrin and chlorophyll metabolism”and “photosynthesis” were the most significantly enriched pathways based on the 109 down-regulated DEGs/DEPs (Additional file 9: Figure S1).

**Table 4:** Correlation of DEGs and DEPs detected in the *T. longibrachiatum* H9-inoculated cucumber plants, compared to untreated plants (+T-B vs. -T-B)

Category	Genes	Proteins
Total numbers of detected genes/Proteins	20,189	4171
Total number of corrected genes/Proteins	3938	3938
Total DEGs/DEPs	3765	922
DEGs/DEPs with corresponding proteins/genes	798	835
DEGs/ DEPs with corresponding DEPs/DEGs	277	277
Corrected DEGs/DEPs with the same expression trend	241 out of 277	241 out of 277
Corrected DEGs/DEPs with the opposite expression trend	36 out of 277	36 out of 277

### Confirmation of qRT-PCR

**Fig. 5:**
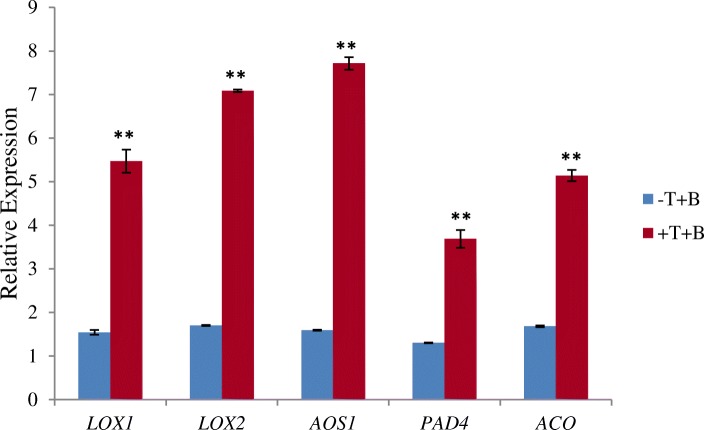
The expression of hormone-related genes was up-regulated in cucumber plants inoculated with *T. longibrachiatum* H9. Among these expressed genes, *LOX1*, *LOX2*, and *AOS1* were associated with JA; *PAD4* was associated with SA; and *ACO* was associated with ET (* and ** indicate significance at *P* < 0.05 and 0.01)

To evaluate the transcriptome results, several genes in the hormone pathways were selected for qRT-PCR analysis in the -T-B and + T-B groups at 96 hp. Ti, such as *LOX1*, *LOX2*, and *AOS1* associated with JA, *PAD4* associated with SA, as well as *ACO* associated with ET. The expression of these genes was significantly up-regulated in the +T-B group, compared to that in the -T-B group (Fig. 5).

### JA and SA content analysis

Based on the transcriptome and proteome results, the key enzymes in JA and SA biosynthesis were up-regulated at both the RNA and protein levels. Therefore, the JA and SA contents in cucumber leaves were analyzed in the -T-B and + T-B groups at 96 hpTi. The SA content increased to 137.29 ± 1.8120 ng/g, and the JA content rose to 151.45 ± 0.0403 ng/g in the +T-B group (Table 5). The JA and SA contents significantly increased in the *T. longibrachiatum* H9-inoculated cucumber plants, compared to that in the untreated plants. These results implied that JA and SA played important roles in regulating the plant response and enhancing plant defense in cucumber plants with *T. longibrachiatum* H9 inoculation.

**Table 5:** The JA and SA contents in cucumber leaves significantly increased after inoculation of *T. longibrachiatum* H9 on the roots

Groups	SA (ng/g)	JA (ng/g)
-T-B	104.24 ± 0.3156	89.48 ± 0.5237
+T-B	137.29 ± 1.8120 **	151.45 ± 0.0403 **

Note: * and ** indicate significance at *P* < 0.05 and 0.01

## Discussion

In this study, we demonstrated that inoculation of *T. longibrachiatum* H9 to the cucumber roots triggered an increased resistance to the leaf pathogen *B. cinerea*, which is characterized by the reduction of the disease incidence. The effect of *T. longibrachiatum* H9 on induction of defense response was monitored through both the transcriptome and proteome analyses. More specifically, further comparison and analysis of transcriptome and proteome data provided a more reliable basis to illustrate the biocontrol mechanism of *T. longibrachiatum* H9 induced systemic resistance against *B. cinerea*. Finally, the following conclusions could be drawn by analyzing our findings and the earlier findings from other researchers.

### Plants respond to *Trichoderma* by hormone production or signaling

It is known that phytohormones play an important role in the regulation of defense responses in plants. Generally, ISR induced by non-pathogen organisms relies on JA signaling, while pathogen-induced SAR is dependent on SA signaling. In recent years, many reported studies have demonstrated that multiple hormones may be involved in shaping ISR [8, 10, 15–23]. Thus, the current view on the specific hormones involved in ISR remains controversial, and the mechanism of ISR seems more complicated. Mathys et al. (2012) found that the SA pathway was a first player in the *T. hamatum* T382-triggered ISR-prime in *A. thaliana*, and SA was synthesized from chorismate rather than phenylalanine. The JA-pathway was not activated at 48 hp. Ti in the *T. hamatum* T382-preinoculated plants without a subsequent infection, and *B. cinerea* challenge caused a clear activation of the JA pathway for ISR-boost [8]. Meanwhile they defined ISR-prime phase as the process of the initial *Trichoderma*-plant interaction and ISR-boost phase as the effects by *Trichoderma* on plant responses after subsequent challenge.

However, Shoresh et al. (2010) mentioned that the presence of non-pathogenic *Trichoderma* may prime the systemic resistance system, which means that the entire pathway may not be turned on constantly, and the plant will react more rapidly or more strongly in a subsequent pathogen infection. Specifically, the priming is not an universal phenomenon in all the *Trichoderma*-plant interactions, for example, *T. harzianum* T22 inoculation caused a constitutive turning on of some PR proteins in maize plants even without any pathogen [15, 48]. Applying specific inhibitors and analyzing the expression of key genes in the JA/ET signaling demonstrated that JA/ET were involved in the protective effect triggered by *T. asperellum* T203 against *P. syringae* pv. *Lachrymans*. Remarkably, the *Lox1*, involved in JA production, was hardly expressed in leaves and only clearly induced in roots with *T. asperellum* T203 inoculation before pathogen infection. In contrast, no difference of SA content in both roots and leaves were observed in the *T. asperellum* T203 pre-inoculated cucumber plants, regardless of whether the plants was infected with *P. syringae* pv. *Lachrymans* or not [10]. Our results showed that both JA/ET and SA production and/or signaling activation were observed as important plant responses in the *Trichoderma*-inoculated cucumber plants. Specifically, some points in our results differed from earlier findings: (i) the key components of the JA/ET and SA pathways were clearly detected in cucumber leaves and significantly up-regulated 96 h post *T. longibrachiatum* H9 inoculation without the subsequent infections, such as LOX (lipoxygenase), AOS (allene oxide synthase) and ACX (acyl-CoA oxidase) associated with JA synthesis, and metK (S-adenosylmethionine synthetase), ACS (aminocyclopropane carboxylate synthase) and ACO (aminocyclopropane carboxylate oxidase) associated with ET synthesis, as well as PAD4 (phytoalexin deficient 4) and PAL (phenylalanine ammonia-lyase) associated with SA synthesis. (ii) The expression of PALs rather than SID2/ICS2 was up-regulated at both gene and protein levels, indicating that SA was synthesized via the phenylalanine pathway rather than from chorismate pathway for *T. longibrachiatum* H9-induced ISR. The discrepancy among different researches suggested that the specific hormones involved may vary and behave differently in different plant-*Trichoderma* interactions, under different conditions and at different time points. However, the two points remain unknown in our study and require further investigation: (i) whether the additional pathogen *B. cinerea* challenge induces a higher plant response in the *T. longibrachiatum* H9-inoculated plants (whether the priming occurs or not), (ii) whether *B. cinerea* induces the plant response by itself or in the *Trichoderma*-mediated manner.

### Plants respond to *Trichoderma* by producing secondary metabolites

The production of secondary metabolites, especially phytoalexins, is an important plant defense strategy against pathogens. Phenylpropanoid compounds are precursors to a wide range of antimicrobial phenolics, including lignins, flavonoids, isoflavonoids and cumarins. These compounds are induced in response to microbial attack and can inhibit the growth of attacking pathogens [3, 49]. The phenylpropanoid pathway is the major source of the production of antimicrobial phenolic compounds. Several genes of the phenylpropanoid pathway have been found to be up-regulated in *A. thaliana* after inoculation with *T. asperelloides* T203, including *PAL1*, *PAL2* and *4CL* [26]. Camalexin, as an important secondary metabolite, accumulated in *A. thaliana* seedlings colonized with *Trichoderma virens* [29]. The biosynthesis of anthocyanins was increased in *A. thaliana* roots after inoculation of *T. hamatum* T382 [8]. In our results, the biosynthesis and metabolism processes of phenylpropanoid, flavonoid, lignin, coumarine, anthocyanin and some other secondary metabolites were activated, which implied that secondary metabolites play important roles for induced plant resistance in the *T. longibrachiatum* H9-inoculated cucumber plants.

Additionally, the antimicrobial activity of lipid transfer proteins (LTPs) in plant defense has also been reported against pathogens in numerous studies, probably due to their ability to interact with biological membranes, which may cause membrane permeabilization [50]. Proteome analysis and various transcriptome studies have also revealed altered expression of LTPs in plants inoculated with *Trichoderma* strains. In our study, lipid oxidation and lipid catabolic processes were also up-regulated in the *T. longibrachiatum* H9-inoculated cucumber plants, which indicated the possible importance of LTPs in the defense against *B. cinerea* in cucumber.

### A hypothetical working process in cucumber plants with *T. longibrachiatum* H9 inoculation

MAMP (microbe-associated molecular pattern)-triggered immunity (MTI) and effector-triggered immunity (ETI) have been reported as two models of innate immunity in plants. *Trichoderma* can produce MAMPs as elicitors to activate plant defense responses. Plants deploy pattern recognition receptors (PRRs) to recognize the elicitors and activate a primary defense response [7, 41, 51–54]. Two MAMP/PRR pairs have been well characterized in *Trichoderma* spp. and plants. The first is ethylene-inducing-xylanase (Xyn2 or Eix) as the elicitor and its corresponding receptor EIX2 [55]. The second is chitin as the elicitor and its corresponding receptor CERK1 (chitin elicitor receptor-like kinase 1) [56]. Successful pathogens will deploy effectors to interfere with MTI and induce ETI which is based on the highly specific interaction of the pathogen effectors and their corresponding NB-LRR class receptors encoded by plant resistance (R) genes. Several transcriptomic and proteomic studies have reported that plant interactions with various *Trichoderma* spp. resulted in the up-regulation of cytoplasmic NBS/leucine rich repeat receptors (NBS/LRRs) [57, 58], suggesting that *Trichoderma* spp. can also induce ETI to activate plant defense.

**Fig. 6:**
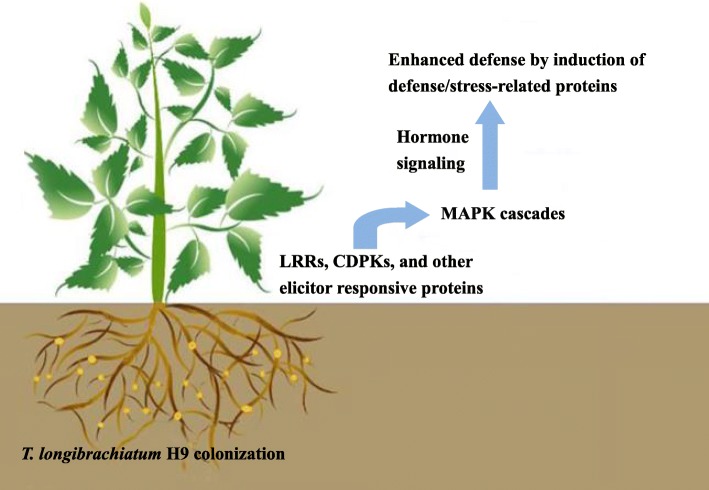
A hypothetical working process in cucumber plants with *T. longibrachiatum* H9 inoculation. LRRs as well as CDPKs and other elicitor-responsive proteins were induced in the *Trichoderma* H9-inoculated cucumber plants to deliver the signal of perception. Subsequently, MAPK cascades were activated to regulate diverse downstream signaling pathways. Furthermore, phytohormone synthesis and signaling (JA/ET and SA) were activated to translate *Trichoderma*-induced signaling into the activation of effective defense responses. Consequently, a whole array of defense/stress-related genes and proteins, e.g. a variety of detoxifying enzymes for ROS scavenging, were up-regulated, thus rendering the cucumber plants more resistant to subsequent *B. cinerea* infection. Along with other defense strategies, the production of secondary metabolites was also employed as an important plant defense strategy

In our results, LRRs, CDPKs, calcium-transporting ATPases and some other elicitor-responsive proteins were up-regulated, which indicated that MTI and ETI immunity systems may exist in the *T. longibrachiatum* H9-inoculated cucumber plants. Moreover, a wide array of plant responses including the activation of MAPK signaling cascades, phytohormone responses, defense/stress-related responses and the production of secondary metabolites, was discovered at the transcription and protein levels. Based on the results, we developed a hypothetical working model in which *T. longibrachiatum* H9 inoculation enhanced plant resistance in cucumber to effectively antagonize *B. cinerea*. Inoculation of *T. longibrachiatum* H9 to cucumber roots resulted in the induction of LRRs and CDPKs and other elicitor-responsive proteins, which are known as specific determinants of the plant immune response to deliver the signal of perception. Subsequently, MAPKs and WRKYs were activated as an early plant response. The induction of the MAPK signaling cascade is known to regulate diverse downstream signaling pathways and convert extracellular signals into intracellular responses [15]. Furthermore, the synthesis and signaling of phytohormones (JA/ET and SA) were activated to translate *Trichoderma*-induced signaling into the activation of effective defense responses. Consequently, a whole array of defense/stress-related genes and proteins, e.g., several types of detoxifying enzymes for ROS scavenging were up-regulated in the cucumber leaves post *T. longibrachiatum* H9 inoculation of the roots, thus rendering the cucumber plants more resistant to subsequent *B. cinerea* attack. Along with other defense strategies, the production of secondary metabolites was also employed as an important plant defense strategy. Finally, these responses would provide cucumber plants with induced resistance to effectively decrease gray mold caused by *B. cinerea* (Fig. 6).

## Conclusions

Root colonization of *T. longibrachiatum* H9 in the outer layer of epidermis effectively promoted plant growth and reduced the disease index of gray mold caused by *B. cinerea* in cucumber. Furthermore, the large-scale, multi-omics datasets indicated that *T. longibrachiatum* H9 inoculation modulated the defense network and resulted in an enhanced defensive capacity in cucumber plants, probably through the activation of signaling pathways associated with the phytohormones JA/ET and SA.

## Additional files

Additional file 1: **Table S1.** The primer sequences for the PCR amplification. (DOC 58 kb)

Additional file 2: **Table S2.** Enriched GO terms based on up-regulated DEGs in the *T. longibrachiatum* H9-inoculated plants in comparison to untreated plants. (XLS 1111 kb)

Additional file 3: **Table S3.** Enriched GO terms based on up-regulated DEPs in the *T. longibrachiatum* H9-inoculated plants in comparison to untreated plants. (XLS 960 kb)

Additional file 4: **Table S4.** Enriched GO terms based on down-regulated DEGs in the *T. longibrachiatum* H9-inoculated plants in comparison to untreated plants. (XLS 439 kb)

Additional file 5: **Table S5.** Enriched GO terms based on down-regulated DEPs in the *T. longibrachiatum* H9-inoculated plants in comparison to untreated plants. (XLS 459 kb)

Additional file 6: **Table S6.** Enriched KEGG pathways based on up-regulated DEGs in the *T. longibrachiatum* H9-inoculated plants in comparison to untreated plants. (XLS 56 kb)

Additional file 7: **Table S7.** Enriched KEGG pathways based on up-regulated DEPs in the *T. longibrachiatum* H9-inoculated plants in comparison to untreated plants. (XLS 51 kb)

Additional file 8: **Table S8.** The list of DEGs/DEPs with the same expression trends. (XLS 134 kb)

Additional file 9: **Figure S1.** The top 20 enriched KEGG pathways based on down-regulated DEGs/DEPs in the *T. longibrachiatum* H9-inoculated plants in comparison to untreated plants (+T-B vs.-T-B). (DOCX 14 kb)
