# Supplementary material for: Involvement of jasmonic acid, ethylene and salicylic acid signaling pathways behind the systemic resistance induced by Trichoderma longibrachiatum H9 in cucumber
Source: BMC Genomics. 2019 Feb 18;20:144. doi: 10.1186/s12864-019-5513-8 (PMC6379975; doi:10.1186/s12864-019-5513-8)
Supplement: Supplementary file 1 — Table S1. The primer sequences for the PCR amplification. (DOC 58 kb) [file 12864_2019_5513_MOESM1_ESM.doc]

**Table S1** The primer sequences for the PCR amplification.

| Primer | Sequences (from 5' to 3') | Reference |
| --- | --- | --- |
| ITS4 | 5'-TCCTCCGCTTATTGATATGC-3' |  |
| ITS6 | 5'-GAAGGTGAAGTCGTAACAAGG-3' |  |
| EF1-728F | 5'-CATCGAGAAGTTCGAGAAG-3' |  |
| TEF1-LLErev | 5'- AACTTGCAGGCAATGTGG-3' |  |
| LOX1-F | 5'-TTGGAGGAAACAAAATCAAAGGGA-3' | This study |
| LOX1-R | 5'-TGGCACTAATGAGTTGGAAAGAAA-3' | This study |
| LOX2-F | 5'-CCTAAGAGCAAACTTGACCCA-3' | This study |
| LOX2-R | 5'-CAATGCCTCAGCAACTGTAAG-3' | This study |
| AOS1-F | 5'-ATCAACCATTCGCAACAAGAG-3' | This study |
| AOS1-R | 5'-AAAGTTCAACCACCATAAGCC-3' | This study |
| ACO-F | 5'-AGAACACATAGCCAGCAAAGG-3' | This study |
| ACO-R | 5'-GGAGATGACGGAGGAAGAAAG-3' | This study |
| PAD4-F | 5'-AGAACAGCAAGCAGAATAGGAC-3' | This study |
| PAD4-R | 5'-CTTGAAGCAATCGTAGTAACCC-3' | This study |
| ACT2-F | 5'-TTGTGCTCAGTGGTGGAACC-3' |  |
| ACT2-R | 5'-ATGGAACCACCGATCCAGAC-3' |  |
