# Supplementary material for: Involvement of jasmonic acid, ethylene and salicylic acid signaling pathways behind the systemic resistance induced by Trichoderma longibrachiatum H9 in cucumber
Source: BMC Genomics. 2019 Feb 18;20:144. doi: 10.1186/s12864-019-5513-8 (PMC6379975; doi:10.1186/s12864-019-5513-8)
Supplement: Supplementary file 9 — Figure S1. The top 20 enriched KEGG pathways based on down-regulated DEGs/DEPs in the T. longibrachiatum H9-inoculated plants in comparison to untreated plants (+T-B vs.-T-B). (DOCX 14 kb) [file 12864_2019_5513_MOESM9_ESM.docx]

**Figure S1** The top 20 enriched KEGG pathways based on down-regulated DEGs/DEPs in the *T. longibrachiatum* H9-inoculated plants in comparison to untreated plants (+T-B vs.-T-B).
